# From Fingerprint Spectra to Intelligent Perception: Research Advances in Spectral Techniques for Ginseng Species Identification

**DOI:** 10.3390/foods15040684

**Published:** 2026-02-13

**Authors:** Yuying Jiang, Xi Jin, Guangming Li, Hongyi Ge, Yida Yin, Huifang Zheng, Xing Li, Peng Li

**Affiliations:** 1 Institute for Complexity Science, Henan University of Technology, Zhengzhou 450001, China; 2 School of Artificial Intelligence and Big Data, Henan University of Technology, Zhengzhou 450001, China; 3 Key Laboratory of Grain Information Processing and Control, Ministry of Education, Henan University of Technology, Zhengzhou 450001, China; 4 College of Information Science and Engineering, Henan University of Technology, Zhengzhou 450001, China

**Keywords:** Panax ginseng, spectral technology, authenticity verification, origin tracing, adulteration detection, quality evaluation

## Abstract

Owing to the high pharmacological relevance and multidimensional quality attributes of *Panax* spp., accurate authentication and quality evaluation of Panax-derived herbal materials remain challenging within traditional Chinese medicine (TCM) quality control systems. Conventional approaches often face trade-offs among analysis speed and throughput, non-destructive measurement, and analytical accuracy, which can limit their suitability for modern, large-scale quality control. This review summarizes recent advances in vibrational and related analytical techniques—infrared (IR) and near-infrared (NIR) spectroscopy, Raman spectroscopy, terahertz (THz) spectroscopy, hyperspectral imaging (HSI), and nuclear magnetic resonance (NMR)—for authentication and quality evaluation of Panax materials. We compare the capabilities of each modality in supporting key tasks, including species authentication, geographical origin tracing, age/cultivation-stage discrimination, and quantitative assessment of major chemical markers, with emphasis on the underlying measurement principles. In general, NIR and HSI are well suited to rapid, high-throughput screening of bulk samples, whereas Raman and NMR provide higher chemical specificity for molecular and structural characterization. To mitigate limitations of single-modality analysis, this review discusses a methodological shift from conventional spectral fingerprinting and chemometric approaches toward model-driven, data-enabled sensing strategies for robust quality evaluation. Specifically, we highlight multimodal data fusion frameworks combined with interpretable machine-learning/deep-learning methods to build robust classification and regression models for quality assessment. This perspective aims to support standardized and scalable authentication and quality evaluation of Panax herbal materials and to facilitate the digitization of quality control workflows for Chinese herbal medicines.

## 1. Introduction

**Figure 1:**
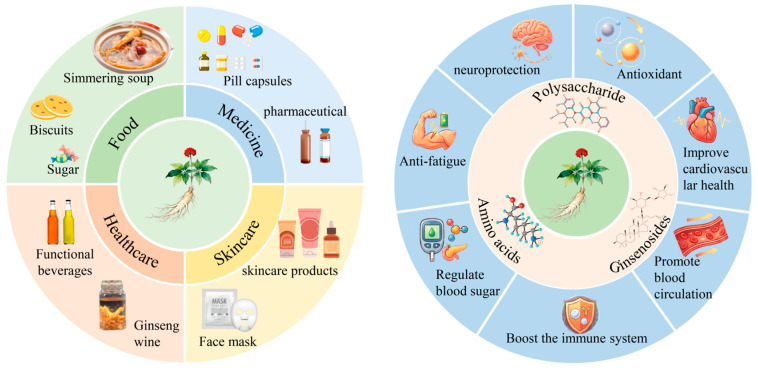
Application areas and pharmacological effects of ginseng plants [12,13,14,15].

The genus Panax comprises economically important medicinal plants with a long history of use in traditional medicine and extensive applications in contemporary commercial markets. Panax species are widely used in traditional Chinese medicine (TCM) and are mainly distributed in temperate regions of East Asia and North America [1,2]. Among commonly traded species, *Panax ginseng*, *Panax notoginseng*, and *Panax quinquefolius* (often referred to as *American ginseng*) account for the majority of cultivated production and international trade [3]. Additional species (e.g., *Panax japonicus* and *Panax vietnamensis*) have also been investigated for their reported pharmacological activities and are used in clinical and commercial contexts [4]. The bioactivity of Panax materials is largely attributed to diverse phytochemicals, particularly ginsenosides, which are commonly treated as key marker constituents. Species-dependent differences in ginsenoside profiles, influenced by genetic background and growing environment, may contribute to differentiated traditional indications and pharmacological activities [5]. For example, *P. ginseng* (often referred to as *Korean ginseng*) is traditionally described as “qi-tonifying” and “spleen-strengthening,” and has been reported to exhibit multiple bioactivities [6,7]. *P. quinquefolius* is primarily associated with lowering blood glucose and blood pressure while aiding in the treatment of various diseases [8,9]; in contrast, *P. notoginseng* (Sanqi) is traditionally indicated for hemostasis and “blood stasis” resolution and has been reported to exhibit analgesic and anti-inflammatory activities, supporting its broad use in pharmaceuticals and health products [10,11] (Figure 1).

With the expansion of international trade in Chinese herbal medicines, concerns regarding the authenticity, quality consistency, and safety of Panax-containing products have increased. Ichim et al. [16] evaluated 507 commercial products labeled as containing ginseng across 12 countries and reported that 76% were authentic, while 24% showed evidence of adulteration or mislabeling. In a recent 2025 review, Orhan et al. [17] expanded the scope to include 911 commercial ginseng products worldwide. Results revealed that the global average adulteration rate remained at 24.7%, with South American markets exhibiting an alarming adulteration rate of 100%—primarily involving species substitution. Furthermore, approximately 48.3% of the functional products tested for chemical adulterants were found to contain illegally added drugs, posing a serious threat to consumer safety. In addition to species substitution, geo-origin mislabeling is an important driver of quality inconsistency in Panax markets. Geo-authentic (“daodi”) production regions are typically associated with characteristic chemical profiles, which may differ from those of non-geo-authentic regions due to environmental and agronomic factors. For example, *P. notoginseng* is commonly associated with Wenshan (Yunnan, China) as a geo-authentic region, and regional differences in ginsenoside content have been reported between Wenshan and other production areas (e.g., Kunming and Yuxi). Supply constraints caused by continuous-cropping challenges and climate-related disruptions may further increase the risk of origin fraud (e.g., mislabeling as “Wenshan *P. notoginseng*”) [18]. Similarly, Wisconsin (USA) is frequently cited as a major production region for *P. quinquefolius* (*American ginseng*), and origin mislabeling has also been reported for *American ginseng* products [19]. Moreover, market value is often linked to claimed growth age and production mode (cultivated vs. wild), which creates incentives for misrepresentation and complicates regulatory enforcement [20].

Conventional identification strategies (e.g., morphology-based examination and targeted chemical assays) have practical limitations for Panax authentication, especially for processed products. Morphology-based identification is highly dependent on expert experience and may be unreliable; processing often removes diagnostic features, further complicating accurate discrimination [21]. Chromatographic methods can deliver high analytical accuracy but may require labor-intensive workflows, longer turnaround times, and destructive sample preparation, which restrict their suitability for high-throughput routine screening [22]. These constraints reduce suitability for high-throughput, on-site, or near-real-time monitoring across the supply chain, including raw material procurement, manufacturing quality control, and distribution [23]. In contrast, spectroscopic and spectral-imaging approaches that capture molecular fingerprints offer rapid, largely non-destructive analysis with reduced reagent consumption, providing a practical route toward digitized quality evaluation of Panax materials and products [24,25]. With advances in chemometrics and machine learning, spectral analysis is progressively transitioning from static fingerprint matching to predictive modeling frameworks that support automated classification, regression, and decision-making [26,27].

This paper provides a comprehensive review of advances in infrared, Raman, terahertz, and hyperspectral imaging methods for authentication and quality evaluation of Panax materials. We discuss modality-specific strengths and limitations with respect to sensitivity, chemical specificity, throughput, and deployability, and outline multimodal data fusion strategies for building next-generation, model-driven sensing frameworks. This review is intended to inform method selection and system design for standardized, scalable quality control of Chinese herbal medicines.

### Literature Search Strategy

A systematic literature search was performed across Web of Science, CNKI, PubMed, and Google Scholar for articles published between 2007 and 2025. Search queries were constructed by combining keywords from three categories: (1) Panax species (e.g., *Ginseng*, *American ginseng*, *Notoginseng*, *P. japonicus*, *P. vietnamensis*); (2) spectroscopic techniques (e.g., FTIR, NIR, Raman, HSI, THz, NMR, UV–Vis, fluorescence); and (3) analysis methods (e.g., machine learning, deep learning, chemometrics). The initial screening was based on titles and abstracts. To be included, studies were required to focus on the quality evaluation or authentication of Panax species using spectroscopy coupled with advanced data analysis. Purely agronomic studies or those lacking chemometric applications were excluded.

## 2. Active Constituents in Ginseng Plants

The chemical constituents underlying the bioactivity of Panax materials are closely linked to their spectroscopic responses, as spectral signals reflect underlying molecular structure and chemical composition. Ginsenosides are characteristic secondary metabolites of Panax and are primarily triterpenoid saponins. Their structural diversity contributes substantially to spectral fingerprints, although measured signatures typically reflect the combined contributions of multiple chemical constituents. Upon electromagnetic irradiation, functional groups in ginsenosides (e.g., C–H, O–H, and C–O bonds and glycosidic moieties) give rise to characteristic vibrational features that can be captured by IR/NIR and Raman measurements; while low-frequency collective modes may be probed in the terahertz (THz) region. These interactions produce modality-dependent absorption and/or scattering signatures that encode chemical and structural information. Therefore, inter-species differences in ginsenoside composition and relative abundance can contribute to differentiated biological activities and yield discriminative chemical fingerprints that support authentication and quality evaluation when combined with appropriate spectral modalities and chemometric models.

According to the aglycone (sapogenin) type, Panax ginsenosides are commonly grouped into protopanaxadiol (PPD)-type, protopanaxatriol (PPT)-type, oleanane (OA)-type, and ocotillol (OT)-type saponins [28]. Chemical-profile differences among Panax species can provide candidate endogenous markers for spectroscopic discrimination, as summarized in Table 1.

Nonetheless, within a given species, ginsenoside/saponin profiles can vary with cultivation age, local microenvironment, and post-harvest processing. This multifactorial chemical variability poses challenges for reliable authentication—particularly for processed products—and highlights the need for modeling strategies that exploit high-dimensional spectral features while accounting for confounders such as origin, age, and processing. Consequently, validated spectral models can support not only species discrimination but also the prediction of cultivation age and the differentiation of processing conditions or quality grades, depending on dataset design and calibration strategy.

## 3. Measurement Principles and Current Applications of Core Spectral Technologies

**Figure 2:**
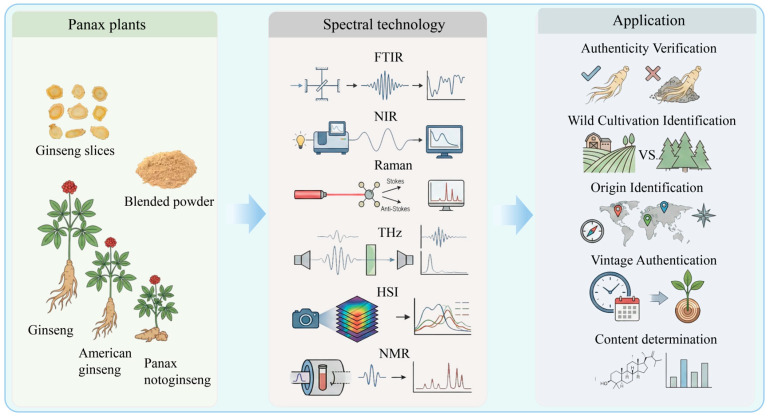
Application of spectroscopic techniques in ginseng plants [4,24,38].

Spectroscopic and spectral-imaging techniques that capture molecular fingerprints enable a largely non-destructive characterization of Panax materials and can be integrated into data-driven workflows for authentication and quality evaluation. The measured signals originate from quantized interactions between electromagnetic radiation and molecular constituents (e.g., ginsenosides), with modality-specific absorption and/or scattering mechanisms. In IR and Raman measurements, functional groups (e.g., C–H, O–H, and C–O bonds and glycosidic moieties) contribute characteristic vibrational features, whereas low-frequency collective modes may also be probed in the THz region [34,35]. These high-dimensional spectra capture chemical variability and may correlate with cultivation age and microenvironmental factors, typically manifested as changes in band positions, shapes, and intensities, when confounders are appropriately controlled [36,37]. With advances in instrumentation and chemometrics/machine learning, spectral analysis is increasingly transitioning from qualitative fingerprint comparison to automated classification and regression models trained on curated datasets, thereby supporting prediction of defined quality endpoints such as species identity, origin, adulteration, marker indices, and processing status (Figure 2).

### 3.1. Infrared Spectroscopy: Functional-Group-Driven Fingerprinting and Quantitative Modeling

Infrared (IR) spectroscopy characterizes samples by detecting transitions in molecular vibrational and rotational energy levels, providing spectral features that reflect functional groups and chemical bond environments. In terms of spectral regions, IR is primarily divided into near-infrared (NIR), mid-infrared (MIR), and far-infrared (FIR), where the specific energy transitions dictate their respective applications. In terms of instrumentation, techniques are mainly categorized into Fourier-transform infrared (FTIR) and dispersive IR spectroscopy. Although FTIR is fundamentally a high-efficiency spectral acquisition technique based on interferometry that theoretically spans from NIR to FIR, in practical analysis and herbal medicine literature, the term “FTIR” is customarily used to specifically refer to MIR spectral analysis. In the context of Panax detection, FTIR (referring to MIR here) measures fundamental molecular vibrations, providing fingerprint spectra with clear chemical significance [39,40,41]. Therefore, it is widely employed for origin traceability, authentication, and structural confirmation (e.g., distinguishing *American ginseng* from *Asian ginseng*). In contrast, near-infrared spectroscopy (NIR) primarily captures overtones and combination bands of X–H groups (e.g., C–H, O–H, and N–H). Whether utilizing dispersive or Fourier-transform (FT-NIR) instruments, NIR technology relies on its deep penetration and rapid, non-destructive acquisition. With reliable calibration models, it is extensively used for quantitative determination and high-throughput screening [42]. For clarity and consistency with established conventions, this study will hereinafter use “FTIR” to denote mid-infrared analysis and “NIR” to denote near-infrared analysis.

#### 3.1.1. FTIR

FTIR applications for Panax authentication have evolved from visual spectral comparison toward complex scenarios assisted by chemometrics and machine learning. Although these domains overlap, they differ significantly in focus and methodology. Chemometrics, originating from analytical chemistry, serves to extract chemically relevant information from data. It specifically addresses issues inherent to spectral analysis, such as collinearity and overlapping peaks. Its primary strength lies in interpretability; researchers often rely on loading plots or regression coefficients to attribute spectral bands to specific chemical bond vibrations. Classic algorithms include Principal Component Analysis (PCA) and Partial Least Squares (PLS). In contrast, machine learning (ML) is a branch of artificial intelligence focused on learning patterns from data to make predictions or decisions. Compared to traditional chemometrics, ML emphasizes predictive accuracy and generalization capability. It excels at modeling highly non-linear and complex relationships, often treating the internal mechanism as a “black box” to prioritize output performance over chemical explanation. Common ML algorithms include support vector machines (SVM), random forests (RF), and deep learning models. Early studies often employed two-dimensional correlation analysis of FTIR spectra (commonly referred to as 2D-IR/2D-FTIR in the literature) and derivative preprocessing to enhance subtle discriminative features. For instance, Lu et al. [43] applied thermal perturbation to generate 2D-FTIR spectra of *American ginseng*, *Asian ginseng*, and *Panax notoginseng*. Analysis of the synchronous 2D-FTIR spectra revealed that the overall spectral patterns exhibit high similarity based on peak positions, suggesting that the chemical constituents of these three materials share generally similar thermal stability. However, the auto-peak curves demonstrated distinct differences in peak position and intensity. Specifically, the strongest auto-peak for *P. notoginseng* appears at 886 cm^−1^, whereas the characteristic auto-peaks for *Asian ginseng* and *American ginseng* are located at 974 cm^−1^ and 971 cm^−1^, respectively. These distinct spectral features allow for the effective differentiation of *P. notoginseng* from *Asian ginseng* and *American ginseng.* Subsequent work increasingly integrated multivariate modeling to address refined identification tasks. Bu et al. [44] reported 100% classification accuracy for distinguishing wild-harvested versus cultivated samples in their dataset using micro-FTIR coupled with discriminant analysis. To reduce reliance on destructive root sampling, Kwon et al. [45] applied multivariate analysis to leaf spectra to classify cultivation age and cultivars.

**Figure 3:**
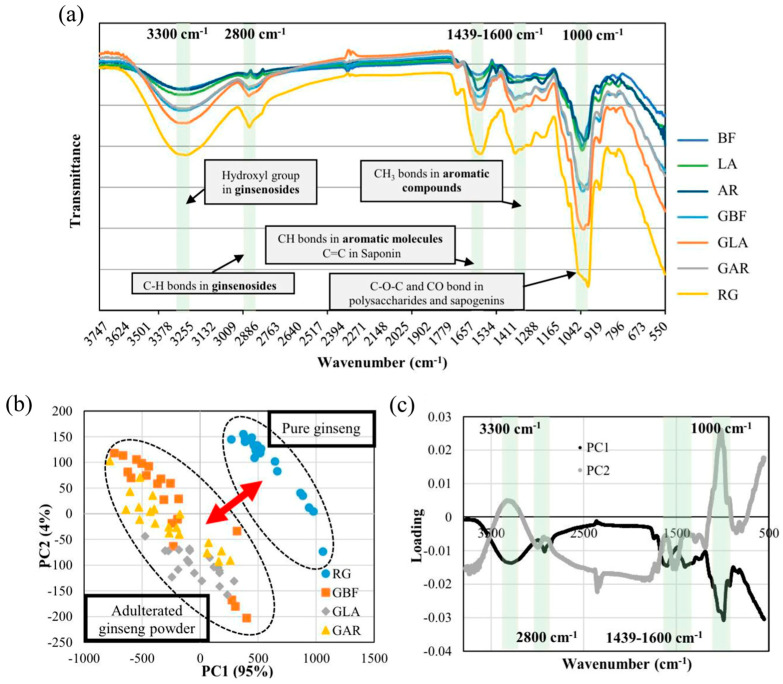
Fourier transform infrared (FT-IR) mean spectra (**a**) from various ginseng powders in the spectral range of 4000-550 cm^−1^, PCA score plot, (**b**) as 2D scatter, and loading plot, (**c**) of the first two principal components of PCA. BF bellflower root powder, LA lance asiabell powder, AR arrowroot powder, RG unadulterated ginseng powder, GLA ginseng powder adulterated with lance asiabell root powder, GBF ginseng powder adulterated with bellflower root powder, GAR ginseng powder adulterated with arrowroot powder [49]. Reprinted from Ref. [49].

More recent studies extended Fourier-transform infrared (FTIR) spectroscopy from single-task authentication to multi-endpoint analysis, including tissue/part differentiation, processing assessment, adulteration detection, and quantitative prediction of marker indices. Li et al. [46] compared starch-, protein-, and calcium oxalate-related signals across different parts of *P. notoginseng*, supporting part-level differentiation. Lee et al. [47] improved the robustness of age and part prediction by optimizing preprocessing strategies (e.g., normalization), the number of Partial Least Squares (PLS) latent variables, and variable-selection settings. Li et al. [48] distinguished sulfur-fumigated versus non-fumigated samples using Fourier self-deconvolution and a back-propagation (BP) neural network. Choi et al. [49] used FTIR to detect adulteration in ginseng powder and to predict saponin-related indices, illustrating FTIR’s utility for combined qualitative screening and quantitative modeling. As shown in Figure 3, the analysis integrated three key aspects: (a) The raw FT-IR spectra highlighted characteristic absorption bands associated with ginsenosides (-OH group at 3300 cm^−1^, C-H bonds at 2800 cm^−1^) and aromatic compounds; (b) The PCA score plot demonstrated effective discrimination, where pure ginseng samples formed a distinct cluster separated from adulterated ones along the PC1 axis, which explained 95% of the total variance; (c) The loading plot confirmed that spectral variations in these specific regions (e.g., 1000 cm^−1^ and 3300 cm^−1^) contributed most significantly to the separation, identifying the chemical basis for discrimination. Lee et al. [50] further developed a multi-factor discrimination model incorporating cultivation age, cultivar, propagation/culture system, and organ type. Collectively, these studies indicate that FTIR-based pipelines can be extended toward multi-endpoint prediction frameworks, which may better support comprehensive quality evaluation of Panax materials in practical workflows.

From a methodological perspective, the performance and transferability of FTIR models depend strongly on preprocessing choices (baseline correction, normalization, derivative methods), instrument-to-instrument variability, sample heterogeneity, and validation design. Therefore, external validation across independent batches and transparent reporting of preprocessing and model parameters are essential for reproducible deployment in industrial and regulatory settings.

#### 3.1.2. NIR

Near-infrared (NIR) spectroscopy refers to the spectral region between visible light and the mid-infrared, where absorption primarily arises from overtone and combination vibrational modes. The same functional groups can exhibit different NIR band positions and intensities depending on the chemical environment and matrix effects [42]. Because NIR spectra are characterized by broad and overlapping bands, reliable analysis typically relies on chemometric preprocessing and multivariate/machine-learning models. Owing to rapid acquisition and deeper penetration, NIR is commonly used for high-throughput screening, online monitoring, and traceability applications involving Panax materials, including discrimination of species and origin, detection of adulteration, and quantitative prediction of marker-related indices (Table 2).

As summarized in Table 2, modeling approaches have expanded from linear methods such as Partial Least Squares Regression (PLSR) to non-linear machine-learning/deep-learning architectures, including convolutional neural networks (CNNs), Long Short-Term Memory (LSTM) networks, and multimodal/transformer variants where applicable. These models can improve non-linear feature extraction from highly overlapped spectra and may enhance prediction of multiple endpoints (e.g., origin, adulteration level, and component indices), provided that calibration transfer, external validation, and control of confounding factors (moisture, particle size, temperature, and batch effects) are adequately addressed. In practice, model robustness for online deployment further benefits from standardized sampling protocols, drift monitoring, and periodic recalibration using representative production batches.

### 3.2. Raman Spectroscopy: Precise Analysis of Microstructures and Isomers

Raman spectroscopy measures the inelastic scattering of monochromatic light and provides vibrational fingerprints with relatively narrow bands and high chemical specificity [67]. Because water typically exhibits weak Raman scattering, Raman measurements can be advantageous for aqueous matrices compared with mid-IR absorption methods. In practice, Raman signal intensity may correlate with analyte concentration, but quantitative analysis generally requires careful control of excitation conditions, fluorescence background, and matrix effects, together with calibration modeling [68].

Raman spectra of Panax materials can reflect regional and processing-related chemical differences through variations in ginsenoside-related bands and other matrix constituents. For example, a Raman band near 980 cm^−1^ has been reported as a discriminative feature for certain “*Chinese ginseng*” samples in a specific dataset [36]. Accordingly, Raman spectroscopy has been explored for rapid, largely non-destructive screening of geographic origin and macroscopic quality endpoints, although robustness across batches and closely related origins should be validated using independent sample sets.

Beyond origin screening, Raman spectroscopy has been applied to refined compositional analysis and authenticity verification. Qu et al. [69] used Raman spectroscopy to differentiate the C-20 epimers (20(R)- and 20(S)-isomers) of ginsenoside Rg3. Although Raman spectroscopy is generally unable to distinguish enantiomers, these diastereomeric forms exhibit distinct steric arrangements at the C-20 position. This structural difference results in measurable variations in Raman features, enabling differentiation even when glycosidic configurations are otherwise similar. Wan et al. [70] compared genuine ginseng with adulterants such as Platycodon grandiflorum, reporting shared bands (e.g., near 1460 cm^−1^ and 1130 cm^−1^) as well as adulterant-specific features; derivative preprocessing was used to enhance class separability. For challenging origin discrimination tasks in which Raman spectra are highly similar (e.g., samples from closely related regions), single-modality Raman may be insufficient. In such cases, multimodal fusion can improve performance. Lin et al. [71] proposed a dual-modality approach that combines laser-induced breakdown spectroscopy (LIBS)-derived elemental fingerprints with Raman-derived molecular fingerprints. Spectral analysis revealed that the LIBS spectra exhibit characteristic atomic emission lines corresponding to metal elements (e.g., Mg, Ca, Na, K) and molecular bands (e.g., C-N, C-C), providing an inorganic profile of the ginseng samples. Complementarily, the Raman spectra captured the vibrational signatures of organic compounds within the 850–1550 cm^−1^ range. By fusing these complementary datasets, the authors reported a significant improvement in origin identification using the CatBoost algorithm, achieving an overall classification accuracy of 99% (with an F1-score > 98%, indicating balanced sensitivity and specificity) in their dataset. This example illustrates how complementary information (elemental vs. molecular) can strengthen classification when validated under well-controlled sampling and cross-batch evaluation protocols.

Raman spectroscopy serves as a vital non-destructive testing technique for identifying traditional Chinese medicinal materials. Leveraging its non-invasive, rapid, and fingerprint-like characteristics, it enables macro-level geographical traceability of ginseng species, distinguishes ginsenoside isomers, and accurately differentiates ginseng counterfeits. Furthermore, when integrated with LIBS and combined with machine learning algorithms, its identification accuracy is significantly enhanced.

### 3.3. Terahertz Spectroscopy: Fingerprint Identification of Molecular Isomers and Fine Structures

Terahertz (THz) radiation lies between microwaves and infrared light and is non-ionizing. THz measurements are sensitive to low-frequency collective vibrations, intermolecular interactions, crystalline or molecular packing features, and intermolecular interactions, which can provide information complementary to IR and Raman spectra [72]. Terahertz time-domain spectroscopy (THz-TDS) is widely used to measure frequency-dependent absorption and refractive index (or transmission) from time-domain waveforms, enabling both spectral fingerprinting and physicochemical characterization [73].

**Figure 4:**
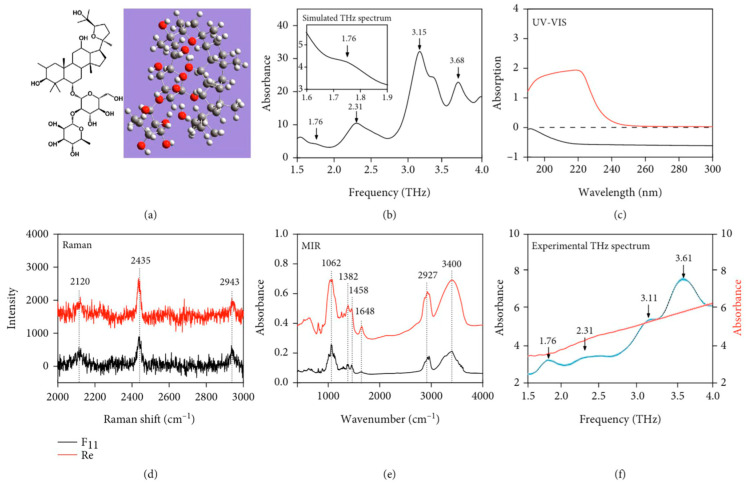
Comparison of different testing methods for 24(R)-pseudoginsenoside F11. (**a**) Molecular formula and theoretical simulation model; (**b**) Theoretical simulation results. Comparative spectra of 24(R)-pseudoginsenoside F11 and ginsenoside Re using (**c**) UV spectroscopy, (**d**) Raman spec-troscopy, (**e**) mid-infrared spectroscopy, and (**f**) terahertz spectroscopy (with error bars from four tests) [74]. Reprinted from Ref. [74].

THz spectroscopy has been explored for authentication using marker compounds and characteristic spectral features. Kou et al. [74] reported the THz spectral features of 24(R)-pseudoginsenoside F_11_ in *P. quinquefolius* (*American ginseng*). As presented in Figure 4, a comparative spectral analysis highlighted the distinct advantage of THz spectroscopy: (a-b) Theoretical simulations predicted specific vibrational modes of F11 in the THz band; (c-e) unlike UV-Vis (where F11 showed no absorption due to its non-conjugated nature), Raman, and MIR spectroscopy (which suffered from significant spectral overlapping with ginsenoside Re), (f) the experimental THz spectra revealed distinct fingerprint peaks (e.g., at 1.76 and 2.31 THz) consistent with simulations, whereas the structurally similar Re exhibited no characteristic features. These findings support the feasibility of THz-based detection and quantification for selected non-conjugated saponins. Importantly, THz methods are best viewed as complementary to established chromatographic assays rather than outright replacements, given differences in sensitivity, selectivity, and standardization requirements.

As applications broadened, THz-TDS combined with machine learning has been reported for geographic origin identification, anatomical part discrimination, and adulteration analysis. Zhang et al. [75] optimized support vector machine-based workflows for geographic identification of *P. notoginseng*. Liu et al. [76] combined THz absorption coefficients with random forest modeling to build an origin classification model for *P. ginseng* with 90% recognition accuracy in their study. Bin et al. [77] reported 100% accuracy for part identification of *P. notoginseng* using SPA-LDA on absorption-coefficient features, and Li et al. [78] integrated THz-TDS with chemometrics for qualitative and quantitative adulteration assessment in *P. notoginseng* powder.

Recent studies have further integrated THz data with deep learning to model non-linear relationships between spectral features and quality endpoints. Zhang et al. [79] reported distinguishable THz features between forest-grown ginseng (MCG) and cultivat-ed ginseng (CG), achieving 97.5% discrimination accuracy and 96% annual grading ac-curacy (for MCG) in their dataset. Gu et al. [80] compared HPLC, UV–Vis, Raman, and THz spectroscopy for detecting ginsenosides R1, Rb1, and Rg1. While HPLC enabled pre-cise quantification via distinct retention times (25.7, 29.0, and 52.4 min), both UV–Vis and Raman spectroscopy faced limitations; the former suffered from noise interference, and the latter showed overlapping peaks at 1467 cm^−1^ and 1530 cm^−1^. Notably, THz spectroscopy successfully identified distinct spectral fingerprints driven by functional group vibrations. R1 displayed specific features in the 7.6–8.9 THz and 10.6–12.1 THz frequency bands, dis-tinguishing it from Rb1 and Rg1, which exhibited broader distributions spanning 7.6 to 12.1 THz. These findings suggest that THz spectroscopy provides separability comparable to HPLC. Furthermore, when coupled with a one-dimensional convolutional neural net-work (1D-CNN), it effectively resolves subtle compositional differences often missed by conventional feature engineering. Wang et al. [81] further proposed a THz-CNN frame-work for non-destructive prediction of cultivation age in forest-grown ginseng.

THz spectroscopy represents an emerging technology for the authentication of traditional Chinese medicinal materials, combining molecular fingerprinting with non-destructive and rapid analysis capabilities. It enables multi-scenario identification, including origin, anatomical part, and adulteration detection. When integrated with algorithms, it demonstrates superior precision in discerning minute variations within medicinal materials and excels in uncovering non-linear relationships compared to techniques such as Raman spectroscopy.

### 3.4. Hyperspectral Imaging: Spatio-Temporal Distribution Perception Through Integrated Mapping and Spectral Analysis

Hyperspectral imaging (HSI) extends multispectral imaging by acquiring a three-dimensional data cube comprising two spatial dimensions and one spectral dimension, in which each pixel contains a reflectance spectrum across tens to hundreds of contiguous narrow bands [82]. By integrating spatial texture with chemically relevant spectral information, HSI enables a transition from single-point measurement to area-based inspection and visualization [83]. HSI has been adopted in food inspection, pharmaceutical quality control, and related applications where non-destructive, high-throughput screening is required [84].

Table 3 summarizes representative HSI studies on Panax materials across classification/traceability, quantitative prediction, and multi-task modeling. Methodologically, many studies report that combining spectral and spatial features improves performance relative to single-feature baselines (e.g., spectral-only or texture-only models). Recent work has also introduced transfer learning and cross-batch adaptation strategies to mitigate dataset shift and enhance model robustness for real-world deployment for practical deployment.

As shown in Table 3, HSI technology is advancing towards greater sophistication and intelligence. Algorithmically, it has evolved from traditional SVM towards deep networks such as TCNA and CNN-GRU, which possess spatiotemporal feature extraction capabilities. Application-wise, it has expanded from single-purpose origin identification to visualization of trace components, multi-task synchronous detection, and cross-scenario breeding. This represents a crucial tool for achieving integrated intelligent perception of both the external appearance and intrinsic properties of ginseng plants.

### 3.5. Nuclear Magnetic Resonance: Chemical Structure Confirmation and Comprehensive Spectral Analysis

Nuclear magnetic resonance (NMR) spectroscopy provides detailed structural information and is widely regarded as a reference method for chemical-structure confirmation. Although limited in portability and typically requiring specialized instrumentation, NMR supports both targeted structural analysis (including 1D/2D experiments) and non-targeted metabolomics for authentication, origin tracing, processing evaluation, and adulteration detection in Panax materials [101]. Table 4 summarizes representative NMR studies.

As summarized in Table 4, NMR technology leverages its non-destructive and high-throughput advantages to establish a deep-sensing system for ginseng plants. This system effectively spans the range from ‘chemical fingerprinting’ to ‘quality phenotyping’. Specifically, ‘chemical fingerprinting’ relies on the structural resolution of NMR to precisely identify endogenous substances, such as the complete signal assignment of core saponins (e.g., Re, Rb1) [110] and the discovery of novel chemical structures [113]. Building upon this, ‘quality phenotyping’ interprets these chemical patterns to discern macroscopic quality attributes. This includes establishing metabolic markers for geographical origin traceability [106], evaluating chemical transformations during processing (e.g., White vs. Red ginseng) [111], and detecting adulteration [107]. Furthermore, advancements in high-resolution techniques such as 2D-NMR and bs-HSQC have effectively overcome signal overlap, significantly enhancing information resolution. Particularly when integrated with multivariate statistics, NMR enables the systematic deconstruction of quality formation mechanisms at the whole-metabolome level, demonstrating its irreplaceable efficacy in resolving complex quality issues.

### 3.6. Other Spectra: Supplementary Perception of Multi-Source Information

Ultraviolet–visible (UV–Vis) spectroscopy and fluorescence spectroscopy are commonly employed as complementary analytical modalities to overcome the limitations of single-technique workflows. Zhong et al. [114] combined UV fingerprint spectra with clustering analysis to differentiate ginseng, *P. notoginseng*, and bamboo-joint ginseng, supporting coarse-grained classification. For adulteration quantification, Bian et al. [115] applied UV diffuse reflectance spectroscopy together with optimized preprocessing and chemometric modeling, reporting correlation coefficients > 0.98 for their best-performing models, These results indicate the suitability of UV-based methods for rapid adulteration screening in *P. notoginseng* powder.

Fluorescence spectroscopy may offer high sensitivity but often requires intrinsic fluorophores or labeling strategies. Because saponins and sugars typically exhibit weak native fluorescence, fluorescence-based workflows may instead exploit co-occurring fluorophores (e.g., tryptophan-containing proteins, phenolic acids, flavonoids). Liu et al. [116] developed a front-face synchronous fluorescence approach to identify starch adulterants in *P. notoginseng* powder, reporting RMSEP ≤ 4% for prediction. Overall, UV–Vis and fluorescence methods are best positioned as low-cost/high-sensitivity supplements within multimodal systems rather than standalone solutions for comprehensive authentication.

Although ultraviolet–visible and fluorescence spectroscopy alone cannot fully meet the core requirements for ginseng species detection, they play an important complementary role within multimodal quality evaluation frameworks: the former demonstrates economic viability through its low cost and ease of operation, proving practical for primary species classification and rapid adulteration screening; the latter achieves highly sensitive identification of adulterants by leveraging the fluorescent properties of protein components. When integrated with chemometrics, these techniques offer lightweight, cost-effective auxiliary solutions for ginseng detection, can be seamlessly incorporated into comprehensive quality assurance systems.

## 4. Comparative Analysis and Ecological Niche of Spectral Technologies

### 4.1. Comparative Analysis of Application Scenarios

By synthesizing comprehensive bibliometric data from the literature review in this study and employing multivariate statistical analysis, functional distinctions and application patterns among different spectral technologies were derived. Addressing the complex quality assessment requirements of ginseng plants, these spectral technologies exhibit significant functional differentiation in detection performance and application scenarios due to their distinct physical response mechanisms. Table 5 provides a detailed comparison of their core principles, advantages and disadvantages, and typical application scenarios.

It can thus be seen that near-infrared (NIR) technology has broad applications in the field of macro-level quality assessment, such as origin tracing and quantitative component analysis. Research by Li et al. [65] demonstrated NIR’s efficacy in undertaking preliminary screening tasks for massive samples within the industry, enabling rapid qualitative and quantitative analysis of large sample volumes. Conversely, Raman spectroscopy and nuclear magnetic resonance (NMR) techniques focus on microscopic material analysis, addressing complex qualitative confirmation challenges through isomer identification and structural validation. Wan et al. [70] validated Raman spectroscopy’s fingerprinting advantages in authenticity verification, while Wang et al. [109] demonstrated NMR’s pivotal role in ginsenoside structural confirmation—a capability unmatched by macro-level spectral techniques. Hyperspectral imaging, leveraging its spectral-image fusion characteristics, has developed unique comparative advantages in vintage identification and visual grading assessments. As reported by Zhao et al. [87] and Chen et al. [89], HSI precisely identifies the growth duration of medicinal materials by leveraging the spatiotemporal characteristics of spectra and textures, compensating for the limitations of single-spectral techniques. This differentiated approach reveals an inherent complementary logic: NIR and HSI provide macroscopic statistical standards, while Raman and NMR furnish microscopic chemical evidence.

### 4.2. Performance Metrics and Ecological Niche Distribution

**Figure 5:**
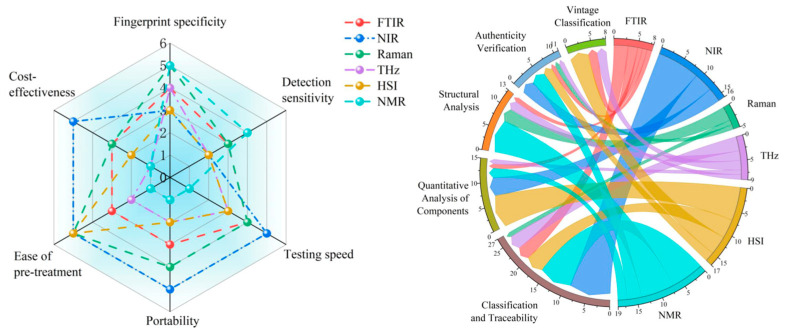
Multidimensional performance evaluation and application niche distribution of spectral technologies. A radar chart illustrating the performance metrics (e.g., sensitivity, cost) of different techniques; a chord diagram visualizing the co-occurrence frequency between spectral techniques and application scenarios. Note: This figure was generated based on multivariate statistical analysis of literature data compiled by this research institute.

Based on the statistical inferences derived from the above data, combined with the multidimensional performance radar chart and application scenario correlation diagram in Figure 5, we can further analyze the ecological niche distribution of each technology within the intelligent perception system.

Through observation and analysis of the charts, it is evident that existing technologies have yet to achieve comprehensive coverage across key dimensions such as specificity, sensitivity, detection speed, and portability. Instead, they have diverged into two distinct camps. The first camp, represented by high-throughput sensing technologies such as near-infrared spectroscopy (NIR) and high-spatial-resolution imaging (HSI), prioritizes detection speed and portability. For instance, studies by Li et al. [60] and Chen et al. [61] demonstrate that NIR combined with chemometric methods enables efficient, low-cost origin tracing, creating favorable conditions for large-scale field screening. However, these technologies exhibit limitations in molecular structural specificity. As noted by Li et al. [64], severe peak overlap in NIR spectra restricts their sensitivity for resolving trace adulterants within complex mixtures. The second category encompasses precision analytical techniques such as nuclear magnetic resonance (NMR), Raman spectroscopy, and terahertz spectroscopy. Their advantages lie in molecular fingerprint specificity and structural resolution capabilities, enabling precise differentiation of isomers. For instance, Qu et al. [69] successfully distinguished isomers of ginsenoside Rg3 using Raman spectroscopy, while Kou et al. [74] demonstrated the sensitivity of terahertz spectroscopy in detecting specific saponin structures like pseudoginsenoside F11. However, limitations imposed by equipment costs and sample preparation requirements hinder their applicability for online real-time monitoring.

In summary, the quality evaluation system for ginseng plants should not be based on the survival of the fittest through single-technology dominance, but rather on the synergistic coexistence of multiple techniques. The macro-level breadth provided by NIR and the micro-level depth offered by Raman/NMR together form the two cornerstones for constructing an intelligent sensing system. Future technological evolution must transcend monomodal thinking, organically integrating the speed of high-throughput sensing with the precision of detailed analysis through data fusion strategies. This will establish a new generation of quality evaluation paradigms that combine macro–micro perspectives and complement dynamic and static aspects.

## 5. Challenges and Prospects

### 5.1. Current Technical Bottlenecks and Limitations

(1) Functional Polarization and Blind Spots in Single-Modality Perception

Many studies rely on a single spectroscopic modality, which often leads to trade-offs between throughput, deployability, and chemical specificity. NIR supports high-throughput screening, but strong band overlap and matrix-related confounding effects can limit selectivity and quantitative accuracy and quantitative performance for trace-level constituents (e.g., low-abundance saponins). Raman spectroscopy provides narrow, chemically specific bands, but performance may be compromised by fluorescence background interference and intrinsically weak scattering signals. THz spectroscopy offers complementary sensitivity to low-frequency collective modes and packing-related differences; however, strong water absorption and environmental/instrumental drift can affect measurement stability. Consequently, single-modality approaches may perform poorly under complex adulteration matrices (e.g., mixtures involving processed residues) or when differences are subtle and distributed across multiple weak spectral features.

(2) The Data Silos Effect and the Crisis of Model Robustness

Many published studies are based on small datasets from single laboratories and lack harmonized protocols for sampling, instrument settings, preprocessing, and ground-truth labeling across regions, harvest years, and devices. Because biological materials exhibit substantial batch effects, models trained on site-specific datasets are prone to overfitting and performance inflation. When deployed to new harvest seasons, instruments, or origins, predictive performance often deteriorates due to domain shift, thereby limiting generalizability, limiting generalization in industrial-scale, cross-scenario applications.

(3) The lack of explanation regarding the mechanism of black-box algorithms

Many recent studies employ end-to-end deep learning pipelines that can improve predictive performance but provide limited interpretability regarding decision drivers. In many cases, emphasis is placed on accuracy metrics rather than establishing mechanistic or chemically grounded links between spectral features and constituent changes (e.g., ginsenosides, polysaccharides) or environmental/processing factors. This limits auditability and regulatory acceptance in high-stakes quality-control settings.

### 5.2. Future Outlook

To address the aforementioned bottlenecks, future spectroscopic research on ginseng plants should focus on paradigm shifts across the following four dimensions to establish a precise, robust, and interpretable intelligent sensing system:

(1) From solitary endeavors to multimodal holistic perception

Comprehensive quality evaluation can be achieved through multimodal data fusion to mitigate limitations of any single technique. For example, NIR can provide rapid bulk screening, Raman can supply chemically specific vibrational fingerprints, THz can add complementary sensitivity to low-frequency collective modes and packing-related differences, and HSI can capture spatial heterogeneity and surface gradients. Data-level or feature-level fusion, coupled with rigorous external validation, can exploit complementary information across modalities. Such fusion may improve detection of trace-level adulteration and selected isomer-related questions and can reduce sensitivity–specificity trade-offs, provided that reference methods, sampling design, and external validation are in place.

(2) From Black-Box Fitting to Explainable Cognitive Intelligence

Future algorithm development should emphasize explainability alongside predictive performance. To achieve this, integrating theoretical insights from quantum-chemical calculations (e.g., DFT for precise band assignment) with empirical data from metabolomics or targeted assays. This combination supports the interpretation of spectral features by linking model-relevant regions to specific chemical constituents. For instance, quantum calculations can theoretically identify which wavenumbers correspond to a specific ginsenoside, while targeted assays confirm its actual concentration in the sample. While model explanation tools (e.g., attention-based attribution, saliency/SHAP-type analyses) can highlight spectral regions contributing to predictions, their stability must be assessed and aligned with such chemically plausible assignments. Establishing mechanistically consistent associations among spectral features, chemical composition, and quality endpoints will significantly improve interpretability and increase confidence in model decisions.

(3) From Offline Detection to Edge-Cloud Collaboration in Ubiquitous Perception

Continued miniaturization and integration of spectroscopic sensors can enable lower-cost handheld devices for at-line screening; smartphone-assisted acquisition and reporting may be feasible for some modalities and use cases. Edge computing paired with cloud databases can support rapid on-site inference with centralized model versioning, drift monitoring, and periodic updates. This edge–cloud collaborative paradigm has the potential to extend quality assessment beyond laboratory environments across cultivation, primary processing, and distribution, enabling faster at-line decision-making where validated.

(4) Standardized Spectral Data Ecosystem

Addressing small-sample and site-specific limitations will require industry-level standards for sampling, acquisition, annotation, and data sharing. Establishing a large-scale spectral database covering multiple species, regions, harvest years, and processing types would provide a foundation for robust modeling and benchmarking. Transfer learning, domain adaptation, and carefully governed incremental updating may mitigate device/year domain shift, but should be evaluated with strict external validation and change-control procedures.

## 6. Conclusions

This paper provides a comprehensive review of recent advances in infrared, Raman, terahertz, hyperspectral imaging, and nuclear magnetic resonance techniques for authentication and quality evaluation of Panax materials. Our synthesis highlights modality-dependent trade-offs arising from different measurement principles, resulting in complementary strengths across various application scenarios. Near-infrared spectroscopy (NIR) and hyperspectral imaging (HSI) are commonly employed for high-throughput screening tasks, while Raman spectroscopy, terahertz spectroscopy (THz), and nuclear magnetic resonance (NMR) offer higher chemical specificity for confirmatory analysis, including targeted isomer and structure-related issues. In data processing, artificial intelligence (AI) strategies, particularly deep learning, have achieved significant success in extracting nonlinear features and enhancing recognition accuracy, surpassing traditional chemometrics. However, their practical application is currently constrained by the “black box” nature of algorithms and the lack of standardized spectral databases. To overcome limitations of single-modality workflows—such as restricted selectivity for trace targets and diminished generalization capabilities during domain transfer—future research should prioritize multimodal data fusion. This should integrate interpretable modeling, standardized data protocols, and rigorous external validation. Integrating spectral information with spatial/texture features and advancing portable/online detection instrumentation holds promise for overcoming the limitations of static fingerprint matching. This approach enables the construction of scalable, model-driven quality control processes for ginseng materials, delivering more robust and interpretable decision support across diverse application scenarios.

## Figures and Tables

**Table 1:** Chemical fingerprint characteristics and pharmacological differences in ginseng species.

Variety	Core Saponin Types	Signature/Distinctive Chemical Fingerprint	Other Associated Photosensitizing Components	Pharmacological Action	Reference
*P. ginseng*	With PPD and PPT types at their core, the range is extensive.	Characteristic marker: Ginsenoside Rf Core constituents: Rb1, Rb2, Rc, Rg1	Pectin polysaccharides (RG-I/RG-II types), polyacetylene compounds	Cardiovascular protection, neuroprotection, antitumor	[29]
*P. quinquefolius*	PPD type predominates, with low PPT content	Characteristic marker: Panax ginsenoside F11 Core constituents: Rb1, Rd, Re	Sucrose, relatively high starch content, polyacetylene compounds	Anti-stress, blood sugar regulation, immune modulation	[30]
*P. notoginseng*	PPT and PPD types predominate	Characteristic marker: Panax notoginsenoside R1 Core constituents: Rg1, Rb1, Rd	Panax notoginsenosides (non-protein amino acids), flavonoids	Cerebrovascular protection, anti-inflammatory analgesia, hemostasis	[31]
*P. japonicus*	Includes OA type, PPD type, and PPT type, with a high proportion of OA type.	Characteristic markers: Bamboo-joint saponin IVa and stipuleanoside R1/R2	Rich in flavonoids, phenolic acids, and high-content polysaccharides	Anti-inflammatory and analgesic, hepatoprotective, and immune-enhancing	[32]
*P. vietnamensis*	Predominantly OT type, with minor amounts of PPT and PPD types	Characteristic marker: Majonoside R1/R2	High volatile oil content Novel dammarane-type saponins	Anti-cancer, liver protection, kidney protection, and neuro-regulation	[33]

**Table 2:** Research overview of NIR in the identification of ginseng plants.

Task Type	Testing Method	Method/Model	Performance Metrics and Key Findings	Reference
Adulteration Quantification	Vis-NIR	CARS-PLSR	Precise prediction of *Panax notoginseng* content through feature band screening, validating the efficacy of full-spectrum feature extraction.	[51]
	NIR	CARS + PLS	Establish standardized quantitative procedures to enable rapid detection of adulterants.	[52]
	NIR + VIS	ANN + LSTM	By introducing LSTM, the detection rate for adulterated *Panax notoginseng* powder reached 100%, demonstrating the advantages of deep learning in processing time-series spectral data.	[53]
	NIR	Dual-branch network	Customized dual-branch network model, training set R^2^ = 0.991, significantly enhancing the depth and breadth of feature extraction.	[54]
Origin Tracing	FT-MIR + NIR	Data fusion	Through advanced data fusion, the identification rate of *Panax notoginseng* origins has been elevated to 98–100%, confirming the necessity of complementary multi-source information.	[55]
	NIR image	ResNet	Converting spectral data into images for input into ResNet, initiating a novel pathway for spectral imaging analysis.	[56]
	FT-MIR + NIR	RF	Multispectral fusion enables rapid, non-destructive origin tracing.	[57]
	NIR + ATRFTIR	Data fusion	Data layer fusion effectively prevents overfitting and enhances classification robustness.	[58]
	NIR + 2D-COS	ResNet	Combining two-dimensional correlation spectroscopy with deep learning to achieve micro-geographical traceability at the county/township level for *Panax notoginseng*.	[59]
	NIR	PLS-DA	Integrating regional data with spectral characteristics to achieve 100% origin classification at low cost.	[60]
	NIR	RSE-LDA	Random Subspace Ensemble Linear Discriminant Analysis Enhances the model’s robustness against interference from anomalous samples Enables classification of ginseng origins	[61]
	NIR + LIBS	Ensemble learning	By integrating atomic emission spectroscopy with molecular vibrational spectroscopy, classification accuracy reaches 99.0%. The complementary nature of multimodal approaches significantly enhances perception precision.	[62]
	NIR	AGOTNet	Proposing a variant of graph convolutional networks, with ginseng origin-tracing accuracy reaching 98.95%, identifying Re and Rb1 as key differential biomarkers.	[63]
Quantitative Classification	FT-NIR	Correlation coefficient method + PLSR	By screening based on characteristic spectral bands, rapid and non-destructive quantification of total saponins in *Panax notoginseng* is achieved, significantly enhancing detection efficiency.	[64]
	NIR	MMTDL	MMTDL simultaneously achieves *American ginseng* traceability and saponin prediction, validating the feasibility of multiple analyses from a single spectrum.	[65]
	FT-NIR + HPLC	PLSR + ML	Achieving simultaneous detection of origin identification (100%) and saponin quantification (RPD > 2.3).	[66]

**Table 3:** Research Overview of HSI Technology in Ginseng Species Identification.

Task Type	Testing Method	Modeling/Algorithms	Performance Metrics and Key Findings	Reference
Classification and Traceability	HSI Reflected Image	MPA-LSSVM	The quality classification accuracy rate reached 95–96.67%, validating its effectiveness in quality grading.	[85]
	HSI + LIBS	multivariate analysis	Combining HSI and LIBS to enhance the robustness of models in authenticating ginseng authenticity and origin identification within complex backgrounds.	[86]
	HSI Reflectance Spectra	RF	For samples aged six years or less, the annual identification rate reached 92.9%, confirming HSI’s capability to capture surface texture and chemical characteristics that evolve over time.	[87]
	HSI	SVM, SAM	Combining spectral and spatial information modeling significantly outperforms single-feature models, enabling precise classification based on growth duration.	[88]
	HSI	FC-CNN	FC-CNN extracts deep features, achieving 100% accuracy in growth year identification, demonstrating the advantages of deep learning in processing high-dimensional data.	[89]
	HSI + Visible Light + X-ray	Ensemble learning	A multi-source heterogeneous data fusion model was constructed, achieving an AUC of 0.997 for origin prediction, demonstrating the immense potential of multimodal fusion technology.	[90]
	HSI	Transfer learning	Introducing transfer learning strategies to address model adaptability across batches, thereby extending application scenarios to breeding selection.	[91]
Quantitative analysis	HSI	AOA-SVR	Combined with AOA prediction of adulteration ratios in *Panax notoginseng* powder, enabling non-destructive visual inspection of blended powders.	[92]
	HSI	BOSS-EO-SVR	BOSS algorithm optimizes wavelength bands, predicting total saponins in *Panax notoginseng* with Rp2 = 0.95.	[93]
	HSI	Chemometrics	Integrating effective wavelength screening enables rapid prediction of trace saponins such as Rg2, overcoming the limitations of HSI detection for low-content components.	[94]
	HSI	TCNA	Six rare saponins predicted to have RPD > 3.0, enabling high-precision simultaneous detection of multiple target components.	[95]
	HSI	CNN-GRU-GPR	Enhancing the ability to predict stability and quantify uncertainty in saponin content.	[96]
	HSI + X-ray imaging	Ensemble learning	Integrating internal density with surface chemical information to achieve a comprehensive assessment of quality.	[97]
	HSI	IRIV-GNDO-ELM	Introducing intelligent optimization algorithms to enhance the Extreme Learning Machine, demonstrating the potential of novel algorithms to improve the performance of traditional models.	[98]
Multi-task collaboration	HSI	MMT1DCNN	Simultaneously achieving traceability of *American ginseng* origins and quantitative analysis of its content, realizing end-to-end intelligent sensing.	[99]
	HSI	1DCNN + Attention	The introduction of a channel attention mechanism enables the visualization of weights to reveal intrinsic correlations between spectral bands, geographical origin, and saponins, thereby enhancing the model’s interpretability.	[100]

**Table 4:** Research Progress of NMR in the Identification of Ginseng Plants.

Task Type	MR Type/Combined Technology	Method/Model	Key Findings and Contributions	Reference
Classification and Traceability	^1^H NMR + isotope	Metabolomics	Distinguishing origins through analysis of carbohydrate and metal element variations, verifying the complementary effects of combining NMR with other techniques.	[102]
	^1^H NMR	Metabolomics	Achieving high-throughput differentiation of closely related species and their origins, laying the foundation for constructing large-scale metabolic fingerprint databases.	[103]
	^1^H NMR	multivariate statistics	Simultaneously distinguishing three reference species from different origins, demonstrating multi-objective classification capability.	[104]
	Non-targeted NMR	Metabolomics	Identifying 52 components and screening for regional markers to achieve metabolic phenotyping traceability across four major production areas.	[105]
Quantitative analysis	LF/HF-NMR	Pattern Recognition	Based on differences in relaxation time, it achieves 100% accuracy when the adulteration ratio is ≥30%, providing a novel rapid screening method for authenticity verification.	[106]
	bs-HSQC + NUS	multivariate statistics	By incorporating non-uniform sampling techniques, testing time is significantly reduced, enabling precise quantification of minute adulterations.	[107]
Component Analysis	^1^H NMR	Multi-step PCA	Revealing significant differences in metabolite lineages provides data support for establishing quality control standards.	[108]
	1D/2D NMR	Structural affiliation	For the first time, complete signal assignment has been achieved for core saponins such as Re and Rb1, establishing a reliable spectral reference library.	[109]
	^1^H NMR	PCA	Distinguish between processed products such as white ginseng and red ginseng, enabling non-destructive evaluation of chemical transformations during processing.	[110]
	NMR + LC-MS/MS	Structural Analysis	Confirm the chemical nature of the unknown peak in black ginseng, thereby rectifying previous misinterpretations of saponin derivatives.	[111]
	2D NMR	Structural assessment	Discovery and characterization of novel saponin structures in Vietnamese ginseng, enriching the chemical fingerprint database of the genus Panax.	[112]
	^1^H NMR	OPLS-DA	It has been confirmed that the understorey cultivation model can significantly enhance saponin content, providing metabolomic evidence for high-quality ecological cultivation.	[113]

**Table 5:** Comparison of commonly used spectroscopic techniques for ginseng species detection.

Spectral Technology	Core Principle	Advantages	Disadvantages	Application Scenarios
FTIR	Characteristic absorption peak analysis of chemical bond types and molecular structures.	Fingerprints offer high specificity, rapid processing, and straightforward pre-treatment.	Water-sensitive, shallow penetration.	Tracing origins, structural analysis, and verifying vintage authenticity.
NIR	C–H, O–H, N–H bond second-harmonic and sum-frequency absorption.	High penetration, portable, suitable for online analysis.	Peak overlap is severe, and model transferability is limited.	Tracing origins, authenticity verification, non-destructive quantification.
Raman	Scattered light analysis of molecular vibrations.	Resistant to water interference, high fingerprint specificity, high resolution.	Fluorescence interference is severe, and the signal is weak.	Structural analysis, traceability, and authenticity verification.
THz	Intermolecular forces, crystal structure and low-frequency vibrational modes.	Non-ionizing and non-destructive, sensitive to crystal structure.	The equipment is costly, and water absorption is intense.	Part identification, traceability, authenticity verification, quantitative analysis of constituents.
HIS	Simultaneous acquisition of spatial imagery and spectral characteristics.	Integration of diagrams and text, rich in information.	Large data volumes, complex modeling.	Quantitative analysis of ingredients, verification of vintage authenticity, traceability.
NMR	Structure of Nuclear Magnetic Resonance Signal Analysis.	Structural analysis is precise, quantitative results are accurate, and the database is extensive.	Pre-processing is cumbersome, detection speed is slow, and the instrumentation is costly.	Tracing origins, complex component identification, authenticity verification.

## Data Availability

No new data were created or analyzed in this study. Data sharing is not applicable to this article.

## Abbreviations

The following abbreviations are used in this manuscript:

IR	Infrared
NIR	Near-Infrared Spectroscopy
THz	Terahertz
HSI	Hyperspectral Imaging
NMR	Nuclear Magnetic Resonance
Vis-NIR	Visible–Near-Infrared Spectroscopy
CARS	Competitive Adaptive Reweighted Sampling
PLS	Partial Least Squares
PLS-DA	Partial Least Squares Discrimination Analysis
PLSR	Partial Least Squares Regression
ANN	Artificial Neural Network
LSTM	Long Short-Term Memory
ResNet	Residual Network
RF	Random Forest
MPA-LSSVM	Marine Predator Algorithm Optimized Least Squares Support Vector Machine
SVM	Support Vector Machine
SAM	Segment Anything Model
AOA-SVR	Arithmetic Optimization Algorithm- Support Vector Regression
bs-HSQC	Band-selective Heteronuclear Single Quantum Coherence
NUS	Non-Uniform Sampling
PCA	Principal Components Analysis
OPLS-DA	Orthogonal PLS-DA
LF/HF-NMR	Low-Field Nuclear Magnetic Resonance
RMSEP	Root Mean Square Error of Prediction
